# Successful treatment of hepatic lymphorrhea by percutaneous transhepatic lymphangiography followed by sclerotherapy using OK-432

**DOI:** 10.1186/s40792-019-0761-z

**Published:** 2019-12-23

**Authors:** Masayuki Kojima, Masanori Inoue, Seiichiro Yamamoto, Toshio Kanai, Seishi Nakatsuka, Motohito Nakagawa

**Affiliations:** 1 Department of Surgery, Hiratsuka City Hospital, 1-19-1 Minamihara, Hiratsuka, Kanagawa 254-0065 Japan; 2 Department of Diagnostic Radiology, Keio University School of Medicine, 35 Shinanomachi, Shinjuku-ku, Tokyo, 160-8582 Japan

**Keywords:** Hepatic lymphorrhea, OK-432, Pancreaticoduodenectomy, Complication

## Abstract

**Background:**

Conventional lymphangiography cannot detect leakage sites of hepatic lymphatic vessels. Percutaneous transhepatic lymphangiography can be used to visualize leakage sites, and once the leakage site has been confirmed, effective sclerotherapy can be performed.

**Case presentation:**

A rare case of intractable hepatic lymphorrhea due to injury of the hepatoduodenal ligament following pancreaticoduodenectomy is reported. Drainage of massive ascites from the drainage tube continued after surgery. Percutaneous transhepatic lymphangiography visualized the intrahepatic lymphatic vessels and the leakage site at the hepatic hilum. An 8-Fr drainage catheter was inserted adjacent to the leakage point under fluoroscopic computed tomography guidance. Repeated sclerotherapy using intraperitoneal administration of OK-432 (picibanil) through the catheter was performed, which exposed the leakage site, and control of the ascites was finally achieved.

**Conclusions:**

To the best of our knowledge, this is the first successful case of detection of a leakage site using intrahepatic lymphangiography, followed by sclerotherapy using OK-432.

## Background

Postoperative lymphatic leakage is an unusual complication after abdominal surgery, although it may occur after a wide range of surgical procedures [1]. Massive ascites is a frequent manifestation and may cause severe malnutrition, which may require additional treatments. Lymph node (LN) dissection is the main cause of lymphatic leakage, and most leakage can be detected by pedal and intranodal lymphangiography. On the other hand, hepatic lymphorrhea associated with injury of the hepatoduodenal ligament is an extremely rare complication, and very few surgeons are familiar with the management of this leakage. Pedal and intranodal lymphangiography is useful for visualizing lumbar and paraaortic lymphatic leakage; however, hepatic lymphorrhea can be visualized using transhepatic lymphangiography [2]. A case of hepatic lymphorrhea that was visualized using percutaneous transhepatic lymphangiography and successfully treated by serial sclerotherapy using OK-432 (picibanil) is presented.

## Case presentation

**Fig. 1:**
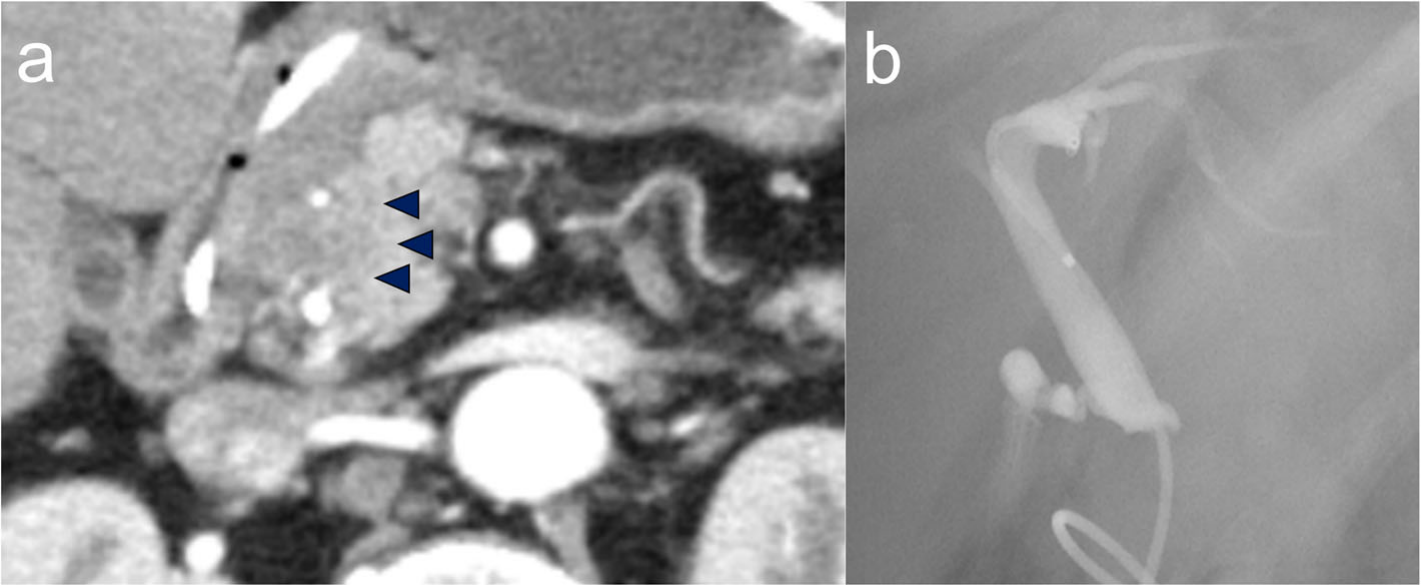
**a** Enhanced CT. Early phase of CT shows a low-density mass approximately 2 cm from the extrahepatic bile duct (black arrowheads). **b** Endoscopic retrograde cholangiography (ERC). ERC was performed and showed a long defect in the lower bile duct, and a tube stent was inserted

A 56-year-old man was admitted to our hospital because of jaundice; he had noticed yellowing of his eyes for 1 month. His medical history included controlled hypertension. A physical examination revealed only jaundice and no ascites. His abdomen was not distended, and no masses were palpable. His laboratory tests showed the following: total bilirubin, 22.0 mg/dL (normal range 0.1–1.0 mg/dL); prothrombin time, 58.6% (80–120%); and serum albumin, 3.7 g/dL (3.9–4.9 g/dL). Contrast-enhanced computed tomography (CT) clearly showed a low-density mass of approximately 20 mm in diameter at the extrahepatic bile duct, with dilation of the intrahepatic bile duct and common bile duct (Fig. 1a). Based on these findings, the diagnosis of extrahepatic bile duct carcinoma was confirmed. Endoscopic retrograde cholangiopancreatography was performed and showed a long defect in the lower bile duct, and a tube stent was inserted (Fig. 1b). However, mild liver dysfunction remained, suggesting chronic hepatic disease. The patient’s Child–Pugh score improved to A, and pancreaticoduodenectomy with LN dissection in the hepatoduodenal ligament was performed after a detailed explanation to the patient about the operation risks and complications associated with chronic liver dysfunction.

**Fig. 2:**
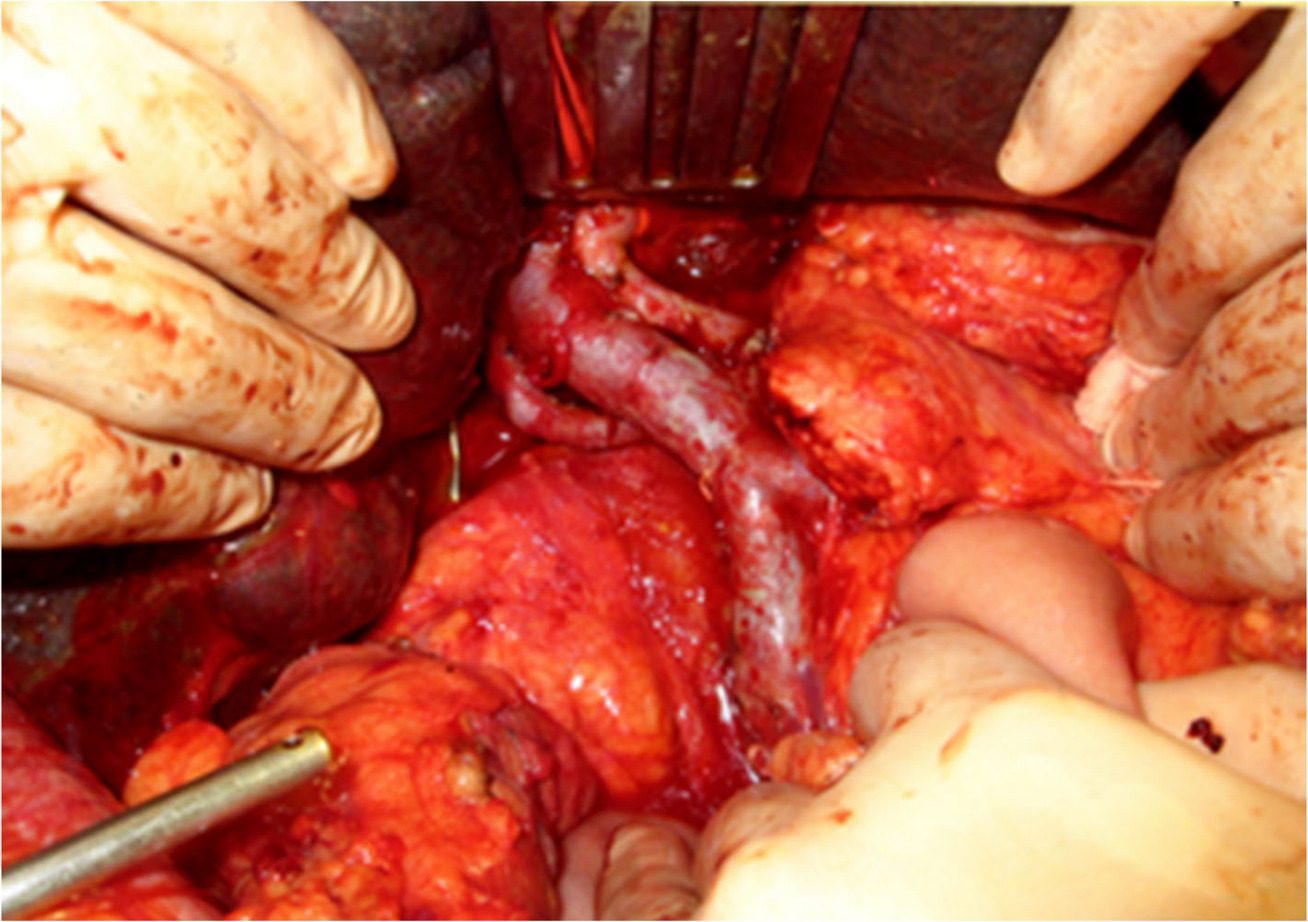
Surgical findings. After the hepatoduodenal ligament was completely skeletonized, the specimen was removed.

**Fig. 3:**
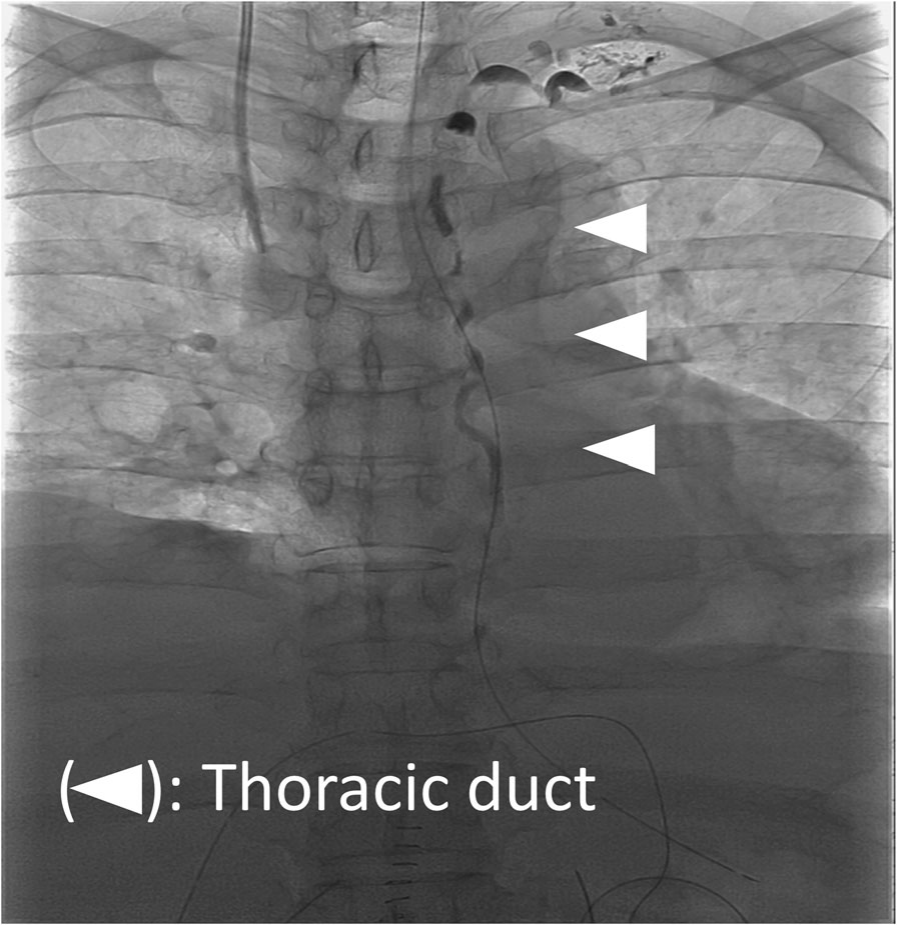
Intranodal lymphangiography performed 2 days after the surgery. In intestinal lymphangiography injected from inguinal lymph nodes, there was no leakage of contrast agent

**Fig. 4:**
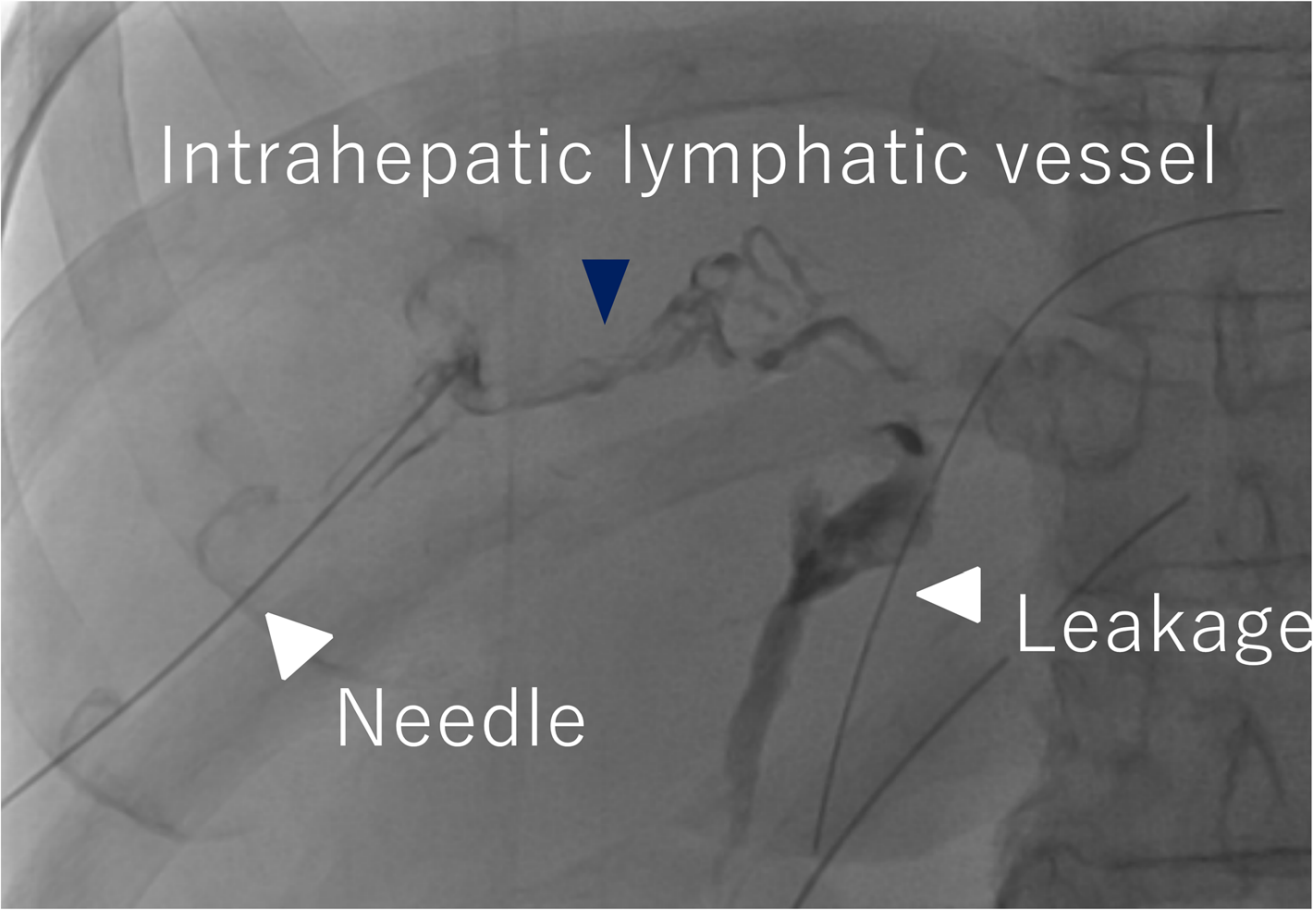
Hepatic lymphangiography. The hepatic lymphatic vessels (black arrowhead) and leakage (white arrowheads) from the hepatic hilum were visualized.

At laparotomy, the liver was jaundiced, and its surface was irregular, indicating cirrhosis. Dilated lymphatic vessels were also found in the hepatoduodenal ligament. Lymphatic vessels were ligated as much as possible during LN dissection of the hepatoduodenal ligament, however, lymphatic fluid continued to leak during surgery. The hepatoduodenal ligament area was sutured again, the leakage of lymph fluid decreased before finishing the operation (Fig. 2). However, more than 3 L of ascites was drained 1 day after the surgery, and lymphatic leakage was suspected. To evaluate the leakage site, intranodal lymphangiography was performed 2 days after the surgery. The hilum of the inguinal LN was punctured under ultrasound guidance using a 60-mm-long, 22-gauge Cathelin needle. Ethiodized oil (Lipiodol 480; Guerbet Japan, Tokyo, Japan) was then manually injected under fluoroscopy. However, no leakage was detected (Fig. 3). Analysis of the ascites showed triglyceride levels < 110 mg/dL, and bacterial culture was negative. Based on the operative findings, hepatic lymphorrhea was suspected. Therefore, percutaneous transhepatic lymphangiography was performed 3 days after the surgery. Glisson’s sheath of the right liver lobe was punctured using a 15-cm, fine (22G) Chiba needle (Hanaco Medical Co., Ltd., Saitama, Japan) under ultrasound guidance, because the hepatic lymphatic system usually runs parallel to the portal venous system. A small amount of 60% Urografin (Schering, Osaka, Japan) was then gently injected while retracting the needle until the lymphatic channels were opacified. After several punctures, intrahepatic lymphatics were visualized, followed by visualization of leakage at the hepatic hilum (Fig. 4).

**Fig. 5:**
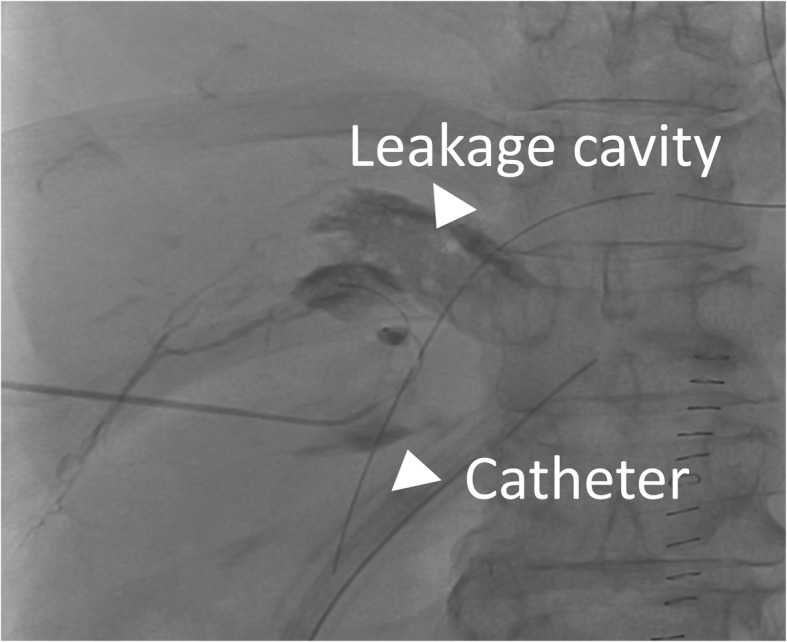
Drainography. Drainography showed the hepatic lymphatics (white arrowheads) retrogradely.

**Fig. 6:**
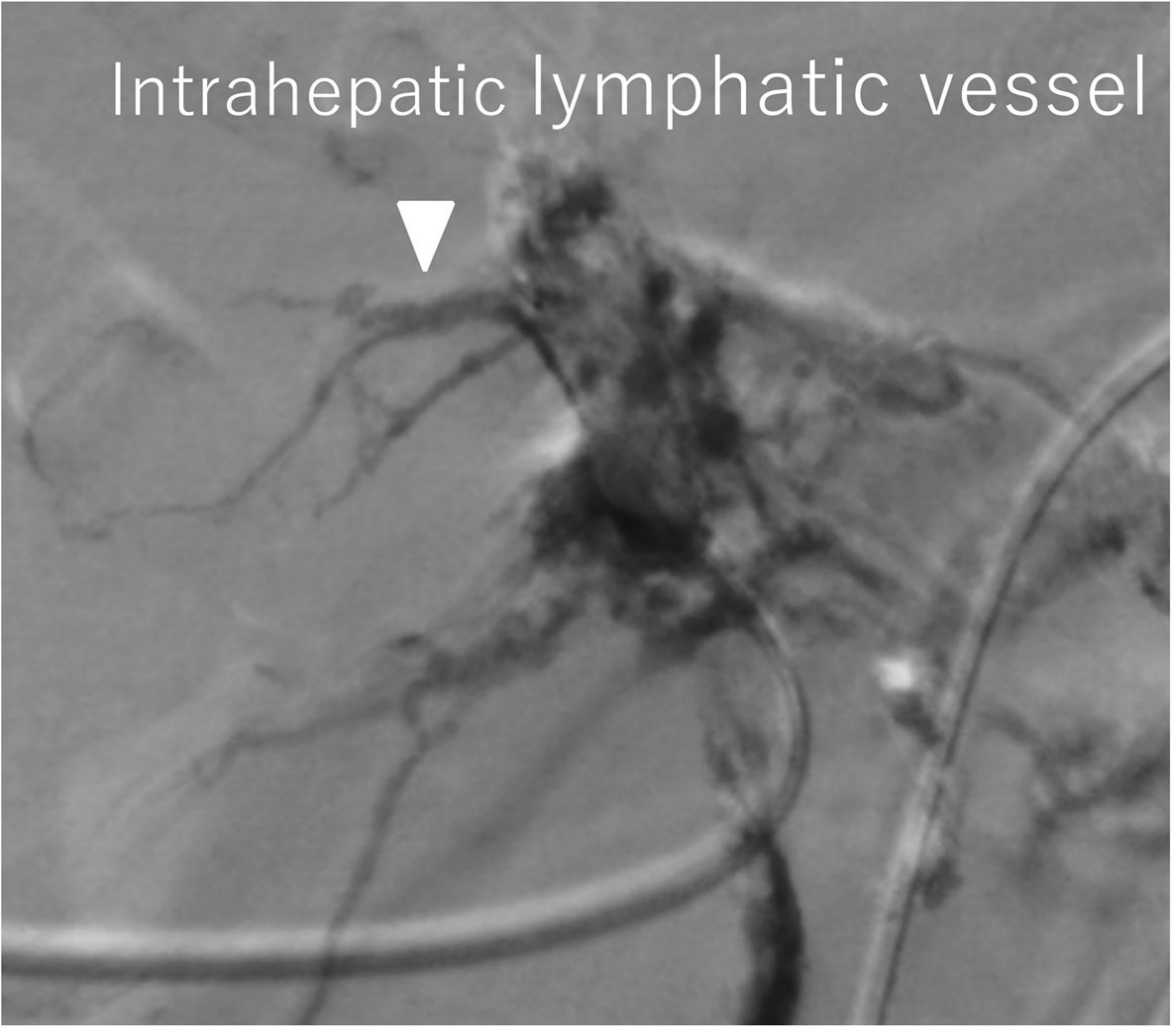
Drainography. OK-432 with contrast agents was injected into the intrahepatic lymphatic vessels to fill the leakage space. As a result, retrograde visualization of the lymphatic vessels gradually disappeared.

The intrahepatic lymphatic vessels through which the microguidewire was advanced were fine and tortuous, and the Chiba needle was easy to retract from the punctured lymphatic vessels. CT detected the accumulation of the contrast material at the hepatic hilum, and the leakage site was confirmed by lymphangiography. Then, a drainage tube (8.5Fr) was inserted into the leakage site under CT fluoroscopic guidance. Fluoroscopic images obtained after the injection of the contrast material through the drainage tube showed stagnation of the contrast material and retrograde filling of the lymphatic vessels (Fig. 5). Sclerotherapy using 2 Klinische Einheit of OK-432 mixed with 4 cc of saline was performed through the drainage catheter retrogradely to ablate the cavity and obstruct the leakage site. The drainage volume from the tube decreased over the next few days, but over 10 L of ascites was drained per day for 6 days after the surgery. Diuretics and albumin were administered, but the ascites could not be controlled. Therefore, OK-432 was repeatedly injected via the tube. The amount of ascites gradually decreased after the fourth injection of OK-432. The drainage tube was replaced to a 6Fr sheath, and glue [25% *n*-butyl cyanoacrylate (NBCA)–ethiodol oil mixture] injection was then performed to fill the leakage space and lymphatic vessels from the microcatheter inserted via the sheath (Fig. 6). On fluoroscopic imaging, retrograde visualization of the lymphatic vessels gradually disappeared. The volume of OK-432 injected was gradually reduced in accordance with the reduction in the size of the cavity. After seven sclerotherapy treatments using OK-432, the patient’s hepatic lymphorrhea was successfully treated without adversely affecting the anastomosis using choledochojejunostomy or pancreaticojejunostomy.

## Discussion

Lymphatic leakage caused by lymphatic injury during abdominal surgery is a rare complication. In most cases, lymphatic leakage results from LN dissection and resolves spontaneously. However, hepatic lymphorrhea, which is leakage from the lymphatic ducts of the liver into the abdominal cavity, is an extremely rare complication, and only a few case reports have been published in the English literature [3–5]. In the present case, a massive amount of hepatic lymphorrhea occurred in the early postoperative period and was resistant to conservative therapies. Sclerotherapy following transhepatic lymphangiography proved to be effective in this case.

The lymphatic system in the peritoneal cavity is roughly divided into three parts: the lumbar, intestinal, and hepatic lymphatic systems [6]. There are two pathways in the hepatic lymphatic system: the ascending pathway along the hepatic veins, and the descending pathway along the hepatoduodenal ligament [7]. Nonchylous ascites consisting mainly of hepatic lymph is usually caused by injury to the lymphatic system from the hepatic hilum to the hepatoduodenal ligament. The liver normally produces a large amount of lymphatic fluid, and it has been estimated that typical lymphatic flow is approximately 0.25 mL/min [8]. Therefore, hepatic lymphorrhea can cause massive ascites. The most reliable surgical treatment for intractable hepatic lymphorrhea is ligation of the injured lymphatic vessels [9]. However, in clinical practice, it is difficult to identify the leakage site (injured lymphatic vessels) in the hepatoduodenal ligament, and only electrocautery of the suspected area of hepatic lymphorrhea or placement of a layer of fibrin glue can generally be performed [5].

It is well-known that the lymph flow is tenfold higher in cirrhotic patients than in healthy individuals due to comorbidities [9]. Even if the lymphatic duct in the hepatoduodenal ligament is damaged during surgery, the lymphatic flow is usually looped around the anastomotic branch, or an anastomosis of the lymphatic vascular channels occurs so that the damaged part is naturally occluded. Therefore, lymphatic leakage becomes problematic in cases of injury to the thoracic duct or cisterna chyli, as well as with the development of dilated lymphatic vessels around them. However, in the present case, pancreaticoduodenectomy did not extend into the thoracic duct and cisterna chyli. Moreover, the ascites was chylous, and triglyceride levels in the ascites were <110 mg/dL. Therefore, it was assumed that the cause of the massive lymphatic injury was not an injury to the thoracic duct and cisterna chyli, but to the lymphatic vessels in the hepatoduodenal ligament. Another factor suggesting hepatic lymphorrhea was that the lymphatic fluid oozed out markedly from the hepatoduodenal ligament during the surgery. In addition, the patient had chronic liver disease. Therefore, the hepatic lymphatic vessels were dilated, and the lymphatic flow was greater than that of normal liver. Thus, the surgically damaged lymphatic vessels were thought not to be spontaneously restored, and intractable hepatic lymphorrhea occurred. Considering these issues, dissection of the LN in the hepatoduodenal ligament in patients with cirrhosis should be performed with extreme caution.

In the present case, diagnostic percutaneous transhepatic lymphangiography was successfully performed, followed by insertion of a drainage catheter into the site of hepatic lymphorrhea. According to previous reports, hepatic lymphatic vessels can be demonstrated incidentally during percutaneous transhepatic cholangiography or portography [10, 11], since intrahepatic lymphatic vessels cannot be visualized by ultrasound, and they are difficult to puncture intentionally. Based on the anatomical information that lymphatic vessels run into Glisson’s sheath, the Chiba needle was advanced parallel to the portal vein and succeeded in puncturing the intrahepatic lymphatic vessels. Since these vessels show characteristic features, such as a beaded appearance and the presence of fine, lucent bands caused by valves, they are easy to recognize. In the present case, the hepatic lymphatic vessels may have been dilated due to cirrhosis. This may be one of the reasons why it was possible to visualize the intrahepatic lymphatic vessels in one session.

Matsumoto et al. [3] reported a case in which transhepatic lymphangiography was used to demonstrate the site of a hepatic lymphatic–peritoneal fistula in a patient who underwent gastrectomy with LN dissection for gastric cancer. They performed transhepatic lymphangiography in the same way as in the present case. They advanced the Chiba needle close to the right portal vein using ultrasound guidance. A 3-mL injection of indigo carmine liquid into the lymphatic vessels colored the ascites draining from the tube slightly blue, thus confirming the fistula. Guez et al. [4] reported a case of successful embolization of hepatic lymphorrhea using the Onyx Liquid Embolic System (Covidien, Plymouth, MN, USA).

In the present case, sclerotherapy was performed because the Chiba needle was spontaneously retracted, and it was difficult to embolize the intrahepatic lymphatic vessels. A single retrograde injection of OK-432 into the injured lymph vessels via a drainage catheter was unsuccessful due to the large amount of lymph flow. There have been several reports on the safety of the adhesion effect of OK-432 in closed spaces. Similar to the present case, the area imaged from the drainage tube was a closed space, and the distance from the anastomosis was sufficient, so OK-432 could be used with caution [12, 13]. Next, the obstructed area was gradually expanded by multiple injections of OK-432, and after seven injections, the leakage site was finally obstructed. There are a few case reports of hepatic lymphorrhea in the English literature [3–5], with approximately 20 cases of hepatic lymphorrhea as a complication of LN dissection in the hepatoduodenal ligament in patients undergoing gastric cancer resection in the Japanese literature [1].

To the best of our knowledge, this is the first report in which the site of hepatic lymphorrhea was visualized and successfully treated using retrograde injections of OK-432 into the injured lymphatic vessels.

It is important to be aware of the rare complications of hepatic lymphorrhea associated with injury to the hepatoduodenal ligament. In cases of persistent hepatic lymphorrhea, transhepatic lymphography followed by OK-432 injection might be a choice.

## Data Availability

Not applicable.

## Abbreviations

CT: Computed tomography
LN: Lymph node
NBCA: *n*-Butyl cyanoacrylate
